# [Retracted] miR-24-3p regulates bladder cancer cell proliferation, migration, invasion and autophagy by targeting DEDD

**DOI:** 10.3892/or.2026.9113

**Published:** 2026-04-06

**Authors:** Guoqiang Yu, Zhaohui Jia, Zhongling Dou

Oncol Rep 37: 1123–1131, 2017; DOI: 10.3892/or.2016.5326

Following the publication of the above article, a concerned reader drew to the Editor's attention that, regarding the cell migration assay experiments shown in Fig. 3B, the ‘miR-24-3p inhibitor’ panels that had been included to represent the T24 and HBC cell lines contained an overlapping section of data, such that data which were intended to show the results of different experiments had apparently been derived from the same original source. Upon performing an independent analysis of the data in this paper in the Editorial Office, it subsequently came to light that several of the western blot data featured in Fig. 3E were strikingly similar to data that appeared in five other papers (one of which was received earlier than this paper at *Saudi Journal of Biological Sciences* with no authors held in common); in addition, cell invasion assay data featured in Fig. 2G were also strikingly similar to data which appeared in a pair of subsequently published papers that too featured no authors in common.

Given the issue of data sharing and the fact that certain of the western blot data had been included in an article that was already been submitted for publication to another journal, the Editor of *Oncology Reports* has decided that this paper should be retracted from the Journal on account of a lack of confidence in the presented data. The authors were asked for an explanation to account for these concerns, but the Editorial Office did not receive a reply. The Editor apologizes to the readership for any inconvenience caused.

